# Correlation Between BI-RADS 4 Subcategories and Histopathological Outcomes in Mexican Women With Breast Lesions: A Retrospective Study of Lesion Laterality and Cancer Incidence

**DOI:** 10.2174/0115734056407923251129144547

**Published:** 2026-02-11

**Authors:** Jose-Emmanuel Castro-Palomares, Irma-Gabriela Sanchez-Rodriguez, Adriana Reyes-Dircio, Linda-Michelle Silva-Lira, Rodrigo-Alejandro Flores-Romero, Ana Alfaro-Cruz, Mauricio Molina-Gonzalez, Ernesto Roldan-Valadez

**Affiliations:** 1 Department of Radiology, General Hospital of México “Dr. Eduardo Liceaga”, 06720, México City, México;`; 2 Breast Imaging Division, Imagen Medica Flosa, 97000, Merida City, México;`; 3 Department of Surgical Pathology, General Hospital of México “Dr. Eduardo Liceaga”, 06720, México City, México;`; 4 Division of Research, Instituto Nacional de Rehabilitación “Luis Guillermo Ibarra Ibarra”, 14389, Mexico City, Mexico;`; 5 Department of Radiology, I.M. Sechenov First Moscow State Medical University (Sechenov University), 119992, Moscow, Russia

**Keywords:** BI-RADS, Breast cancer, Mammography, Ultrasound, Histopathology, Malignancy risk, Breast lesions, Diagnostic imaging

## Abstract

**Introduction:**

BI-RADS category 4 is subdivided into 4A, 4B, and 4C to convey increasing levels of suspicion for malignancy, yet reported predictive performance varies across settings. We evaluated how BI-RADS 4 subcategories relate to histopathological outcomes in Mexican women and explored whether age, lesion laterality, and imaging findings provide additional context for malignancy risk.

**Materials and Methods:**

This retrospective cross-sectional study included 173 women with BI-RADS 4 lesions evaluated by mammography and/or ultrasound, with subsequent histopathological confirmation. Cases were identified at the Hospital General de México between January 2023 and May 2024. Associations between BI-RADS subcategory and malignancy, as well as age, lesion laterality, and selected imaging features, were examined using chi-square tests and one-way ANOVA.

**Results:**

Among 173 patients, 41.6% had BI-RADS 4A lesions, 35.8% had 4B lesions, and 22.5% had 4C lesions. Malignancy rates increased across subcategories: 7.5% (4A), 40.0% (4B), and 85.0% (4C) (*p* < 0.001). Mean age rose with BI-RADS level (42.1, 47.8, and 55.3 years for 4A, 4B, and 4C, respectively), although this trend did not reach statistical significance (p = 0.063). Nodules were the most frequent imaging finding (83.2%), and fibroadenoma was the most common benign diagnosis. Left-sided lesions were more frequently malignant (p = 0.034).

**Discussion:**

In this cohort, BI-RADS 4 subcategorization separated malignancy risk in a clinically meaningful way. Lesion laterality showed an association with malignancy and may merit further evaluation as an adjunct variable in imaging audits.

**Conclusion:**

BI-RADS 4 subclassification corresponded to progressively higher malignancy rates, supporting its use for risk communication and biopsy prioritization. Incorporating histopathological outcomes with basic demographic and anatomic variables, such as laterality, may further strengthen local risk assessment strategies.

## 1 INTRODUCTION

Mammography (MMG) remains the cornerstone of breast cancer screening, but modern practice increasingly combines MMG with Ultrasound (US) and, when indicated, digital breast tomosynthesis to improve lesion detection and characterization, particularly in women with dense breasts [1, 2]. These advances have also supported broader use of minimally invasive, image-guided percutaneous biopsy, which has largely replaced diagnostic surgery in many settings [3, 4].

Even with improved imaging, false-positive findings remain common. Prior reports suggest that only a small fraction of biopsied “suspicious” lesions are ultimately malignant, underscoring the need for consistent interpretation frameworks to guide decision-making and patient counseling [5].

To standardize reporting, the American College of Radiology developed BI-RADS. Category 4 is particularly relevant because it denotes suspicion that warrants tissue diagnosis and is subdivided into 4A (low, 2–10%), 4B (moderate, 10–50%), and 4C (high, 50–95%) to better communicate risk and support clinical triage [6, 7].

However, agreement between BI-RADS 4 subcategories and histopathological outcomes has been inconsistent across institutions and populations. Reader variability, technical factors, and patient characteristics may influence performance, and evidence from Latin American cohorts remains limited [8-11].

We therefore assessed the correlation between BI-RADS 4A–4C subcategories and histopathological outcomes in patients evaluated at a high-volume public hospital in Mexico, and we examined whether age, lesion laterality, and selected imaging features were associated with malignancy. Our goal was to provide locally relevant data that can inform diagnostic audits and practical risk stratification in routine care.

## 2 METHODS

### 2.1 Study Design

This retrospective, observational, cross-sectional study was performed at the Hospital General de Mexico “Dr. Eduardo Liceaga.” The study followed the principles of the Declaration of Helsinki and applicable national biomedical research guidance from the Secretaría de Salud (Mexico). Reporting was organized according to STROBE recommendations for cross-sectional studies.

### 2.2 Population and Sample

We included female patients who underwent mammography and breast ultrasound between January 9, 2023, and May 14, 2024, with imaging classified as BI-RADS 4A, 4B, or 4C and subsequent histopathological confirmation. The final dataset comprised 173 cases meeting all inclusion and exclusion criteria.

All patients underwent full-field digital mammography as part of routine evaluation. In women with dense breasts or equivocal findings, digital breast tomosynthesis was additionally performed at the radiologist’s discretion in line with institutional policy. Together, these modalities supported lesion characterization and selection of the most appropriate image-guided biopsy approach.

### 2.3 Sample Size Calculation

The sample size was calculated based on the formula for estimating a population proportion:

n = Z^2^ × P(1 - P) / Δ^2^ (Equation 1 [12])

Where:

- Z represents the confidence level (95%) with Z^2^ = 3.842,

- P is the expected proportion in the population (7%, or 0.07), and

- Δ is the desired precision (7%, or 0.07).

Substituting these values into the formula:

n = (3.842 × 0.07 × (1 - 0.07)) / (0.07^2^)

n = (3.842 × 0.07 × 0.93) / 0.0049

n = 51

Although the minimum sample size was 51 cases, 173 eligible patients were included to improve statistical power and external validity.

### 2.4 Sampling Method

We used a non-probabilistic consecutive sampling method, selecting all eligible patients from the institutional imaging and pathology databases during the study period. This method facilitated the comprehensive capture of all qualifying BI-RADS 4 cases within the timeframe.

### 2.5 Inclusion and Exclusion Criteria

- **Inclusion Criteria:**
  - Female patients with imaging findings categorized as BI-RADS 4A, 4B, or 4C.
  - Histopathological confirmation following biopsy.
- **Exclusion Criteria:**
  - Patients classified outside the BI-RADS 4 subcategories.
  - Incomplete clinical, imaging, or pathology data.
- **Elimination Criteria:**
  - Cases with no histopathological diagnosis available.

### 2.6 Variables and Operational Definitions

The study analyzed demographic, imaging, and histopathological variables:

- **Age:** Measured in years at the time of diagnosis (quantitative, continuous).
- **BI-RADS Subcategory:** 4A, 4B, or 4C classification based on MMG and US (qualitative, ordinal).
- **Lesion Characteristics:** Type of abnormality, including nodules, microcalcifications, amorphous calcifications, axillary lymph nodes, or collections (qualitative, nominal).
- **Lesion Laterality:** Location of the lesion (left, right, or bilateral) (qualitative, nominal).
- **Histopathological Diagnosis:** Classified as benign (*e.g*., fibroadenoma, mastitis) or malignant (*e.g*., DCIS, invasive carcinoma) (qualitative, nominal).
- **Biopsy Type:** Method used for tissue sampling (Core Needle Biopsy (CNB), fine needle aspiration, or stereotactic biopsy, (qualitative, nominal).

### 2.7 Data Collection and Management

Data were extracted from the hospital’s radiology and pathology databases using a structured form (Annex 1). All personal identifiers were removed, and patients were assigned anonymized codes. The dataset was curated and stored securely by the research team for subsequent analysis.

### 2.8 Statistical Analysis

Descriptive statistics were calculated for all variables. Categorical variables (*e.g*., BI-RADS subcategory, lesion type, histopathological diagnosis, laterality) were summarized using frequencies and percentages, while continuous variables (*e.g*., patient age) were described using mean, standard deviation, and range.

To evaluate the association between BI-RADS subcategories and histopathological outcomes, as well as between lesion laterality and malignancy status, Pearson’s chi-square test was used. For continuous variables, specifically to assess differences in mean Age across the BI-RADS 4A, 4B, and 4C categories, a one-way Analysis Of Variance (ANOVA) was performed. Statistical significance was set at *p* < 0.05 for all comparisons.

Data were analyzed using IBM SPSS Statistics version 27, and visual representations (bar charts and histograms) were produced in Microsoft Excel and Python to enhance the interpretability and clarity of the findings.

### 2.9 Ethical Considerations

The study was approved by the Research Ethics Committee of the Hospital General de Mexico (reference number DECS/JPO-CT-2821-2025). As a retrospective analysis using anonymized data, informed consent was waived in accordance with institutional guidelines. The study posed no additional risk to patients beyond routine clinical procedures.

## 3 RESULTS

### 3.1 Patient Demographics

A total of 173 female patients were included. Mean age was 45.8 years (SD 12.7). Mean age increased across BI-RADS subcategories, 42.1 years (SD 10.2) in 4A, 47.8 years (SD 12.5) in 4B, and 55.3 years (SD 14.0) in 4C (Table **1**). The difference did not reach statistical significance (ANOVA, p = 0.063).

### 3.2 Lesion Characteristics

Nodules were the most common imaging finding (144/173, 83.2%). Amorphous calcifications (3.5%) and microcalcifications (2.9%) were less frequent, and axillary lymphadenopathy and breast fluid collections were each observed in 5.2% of cases (Table **2**). Lesion type was not significantly associated with BI-RADS subcategory (chi-square test, p = 0.212).

### 3.3 Histopathological Diagnoses

Benign histopathological diagnoses accounted for 58.4% of cases, most commonly fibroadenoma (19.1%), stromal fibrosis (16.2%), and mastitis (13.9%). Among malignant lesions (41.6%), ductal carcinoma *in situ* (11.0%), invasive ductal carcinoma (10.4%), and invasive lobular carcinoma (6.9%) were most frequent (Table **3**).

### 3.4 BI-RADS Categories and Malignancy Correlation

For the subgroup with complete matched imaging and pathology used in this analysis, BI-RADS subcategories were distributed as BI-RADS 4A (n = 40), 4B (n = 30), and 4C (n = 30) Figs. (**1**-**3**). Malignancy rates increased across categories: 17.5% in 4A, 70.0% in 4B, and 86.7% in 4C (Table **4**).

Histopathological outcomes differed significantly across BI-RADS subcategories (χ^2^ = 34.94, df = 2, *p* < 0.001), supporting the discriminatory value of BI-RADS 4 subcategorization for malignancy risk in this cohort.

### 3.5 Lesion Laterality

Laterality analysis showed a predominance of left-breast lesions (83.5%), with fewer right-sided (11.5%) and bilateral (5.0%) cases. Left-sided predominance persisted across BI-RADS subgroups (Table **5**). Laterality was associated with malignancy (chi-square test, p = 0.034).

## 4 DISCUSSION

### 4.1 Clinical Relevance of BI-RADS 4 Subcategorization

BI-RADS 4 subcategorization (4A–4C) was introduced to provide a more granular estimate of malignancy risk and to support decisions about biopsy and follow-up. In our cohort, malignancy rates increased from 4A to 4C and were broadly consistent with the risk ranges described in the ACR BI-RADS^®^ Atlas [13, 14].

The separation in malignancy rates across 4A–4C in our data argues for maintaining these subcategories in routine reporting. Although some authors have suggested simplifying category 4 because of overlap between sublevels [10], our results showed clear gradients that can help prioritize biopsy and structure patient counseling, particularly in settings where access to procedures may be constrained [8, 15].

### 4.2 Imaging and Histopathological Correlation

As expected, most 4A lesions were benign, with fibroadenoma and benign phyllodes tumors appearing frequently. In 4B, benign and malignant diagnoses were mixed, including low-grade invasive ductal carcinoma, whereas 4C lesions were predominantly malignant (*e.g*., higher-grade invasive ductal carcinoma and invasive lobular carcinoma), with relatively few benign outcomes.

Our statistical dataset captured histopathology at the diagnosis level (Table **3**) without additional tumor-level variables (*e.g*., size, receptor status, lymphovascular invasion). To strengthen the imaging–pathology narrative within these constraints, we included representative gross and microscopic images Figs. (**4**-**6**), which illustrate the spectrum of findings across BI-RADS 4 subcategories [2, 15-18].

### 4.3 Radiological Characteristics

Most suspicious findings in this series were nodules rather than microcalcifications. Similar patterns have been reported in Mexican cohorts and in other Latin American and Asian settings, which may reflect population characteristics, referral pathways, and the frequent combined use of US with MMG in diagnostic workups [8, 19].

By contrast, studies based on screening populations have sometimes reported a higher proportion of calcification-driven BI-RADS 4 assessments, particularly in younger women [9-11]. The imaging mix in our hospital may therefore reflect local practice and case-mix rather than a universal phenotype.

Regional or ethnic variations in breast density and imaging access might influence the radiological presentation of breast lesions [8]. When ultrasonography is used as a first-line modality in combination with mammography, as in our institution, there may be a higher detection rate of nodular abnormalities [15, 16].

Use of full-field digital mammography, supplemented by tomosynthesis when indicated, may also influence the distribution of reported findings by improving visualization of subtle architectural distortion and margin features [14, 20, 21].

Whether BI-RADS 4 should remain subdivided is still debated. In our practice, keeping 4A–4C has practical value because it helps convey urgency and supports shared decision-making, even if some overlap between adjacent subcategories is unavoidable [11, 22].

Overall, our results support retaining BI-RADS 4 subcategories while emphasizing the need for ongoing reader training and periodic local audit to limit interobserver variability [22, 23].

### 4.4 Laterality and Malignancy

Laterality is not routinely emphasized in many imaging reports, yet we observed a marked predominance of left-sided lesions and a significant association between left-sided location and malignancy. The biological basis of such asymmetry remains uncertain, but hypotheses include anatomic differences, breast-size variation, and detection-related factors [18, 19, 23, 24].

Prior epidemiologic work has described small but persistent laterality differences in breast cancer incidence, although the direction and magnitude vary across studies [17, 25]. At minimum, our findings suggest that laterality is a variable worth tracking in institutional audits and in future population-based analyses [23, 24].

### 4.5 Clinical Implications

Taken together, our results indicate that BI-RADS 4 subcategorization is most informative when interpreted alongside basic demographic and anatomic context. If confirmed in other cohorts, the laterality signal observed here could inform how institutions monitor diagnostic performance and counseling practices [26, 27].

### 4.6 Study Limitations

This single-center, retrospective design limits generalizability. Although the sample size supported the main comparisons, analyses by specific histologic subtype were constrained by small cell counts. We also did not measure interobserver variability in BI-RADS assignment, which may contribute to classification differences reported in the literature [19].

Because the pathology database provided diagnosis-level information only, we could not evaluate additional prognostic variables (*e.g*., tumor grade, margins, receptor status). These data limitations restrict deeper imaging–pathology modeling, but the illustrative pathology figures Figs. (**4**-**6**) support the descriptive comparisons across BI-RADS subcategories.

### 4.7 Future Directions

Future multicenter prospective studies should validate these findings, quantify reader agreement for BI-RADS 4 subcategories, and test whether advanced tools (*e.g*., contrast-enhanced MMG or AI-assisted decision support) can reduce subjectivity. Additional work on laterality mechanisms and on patient-centered outcomes (*e.g*., anxiety and decision-making) would complement the present results and may help refine local screening and triage strategies.

## CONCLUSION

In this cohort of Mexican women, BI-RADS 4 subcategorization (4A–4C) corresponded to progressively higher malignancy rates and showed robust discriminatory performance. The predominance of nodular findings, the histopathological spectrum across subcategories, and the observed laterality pattern highlight the value of periodic local audits. In resource-variable environments, retaining BI-RADS 4 subcategories may support biopsy prioritization and reduce unnecessary procedures when combined with clinicodemographic context.

## Figures and Tables

**Fig. (1):**
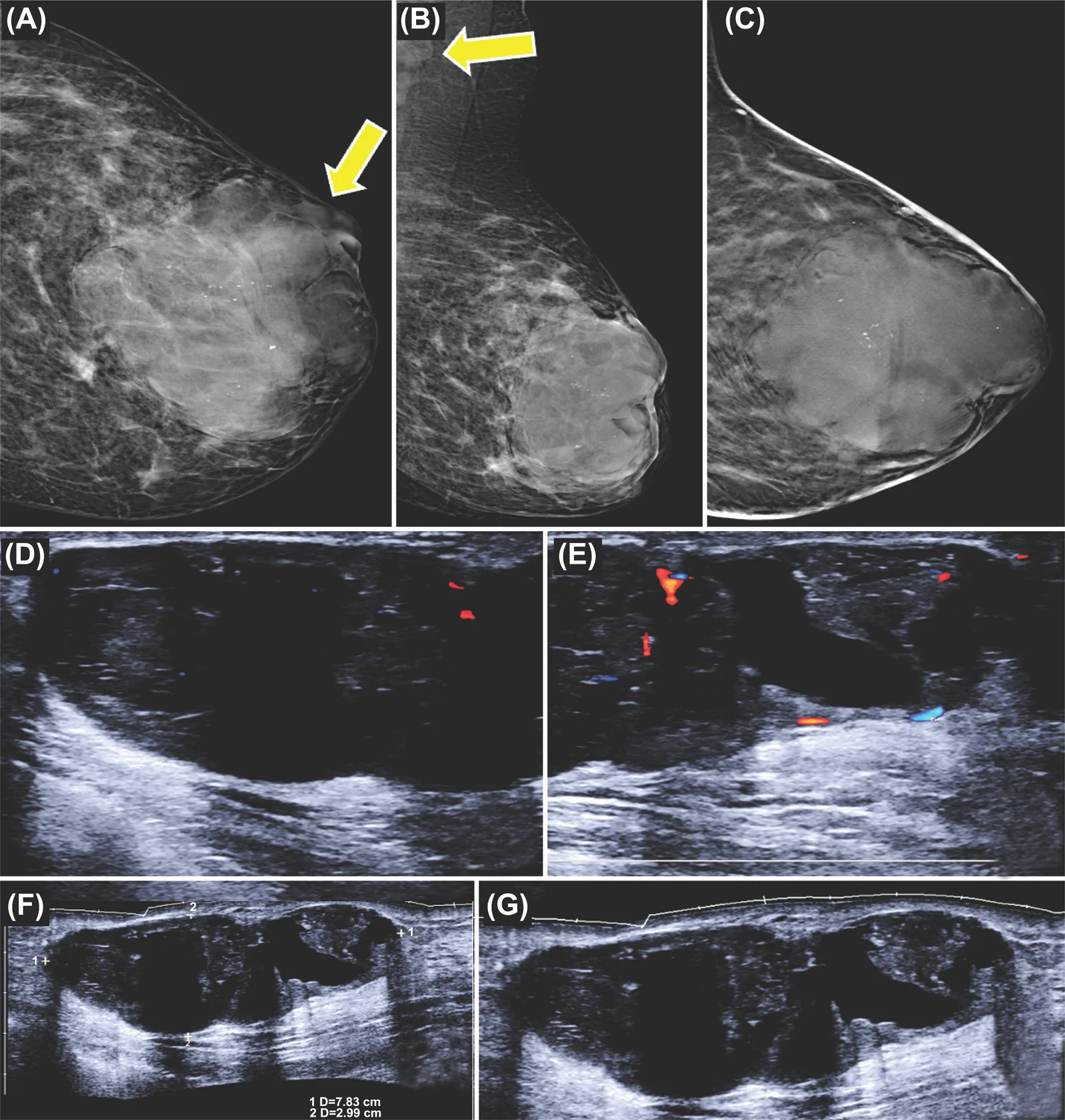
BI-RADS 4A lesion. Craniocaudal mammographic view (**A**) of the left breast shows an oval, circumscribed nodule with similar density to surrounding tissue, associated with heterogeneous calcifications and cutaneous extension, located centrally in the anterior-middle third (yellow arrow). Medio-lateral oblique view (**B**) shows preserved axillary lymph nodes (yellow arrow). Tomosynthesis (**C**) highlights architectural distortion more clearly. US images (**D**, **E**) reveal a complex, solid-cystic, oval mass parallel to the skin, with central and peripheral Doppler flow. The mass measures approximately 78.3 × 29.9 mm in its longest axes (**F**), and additional grayscale imaging confirms its morphology (**G**).

**Fig. (2):**
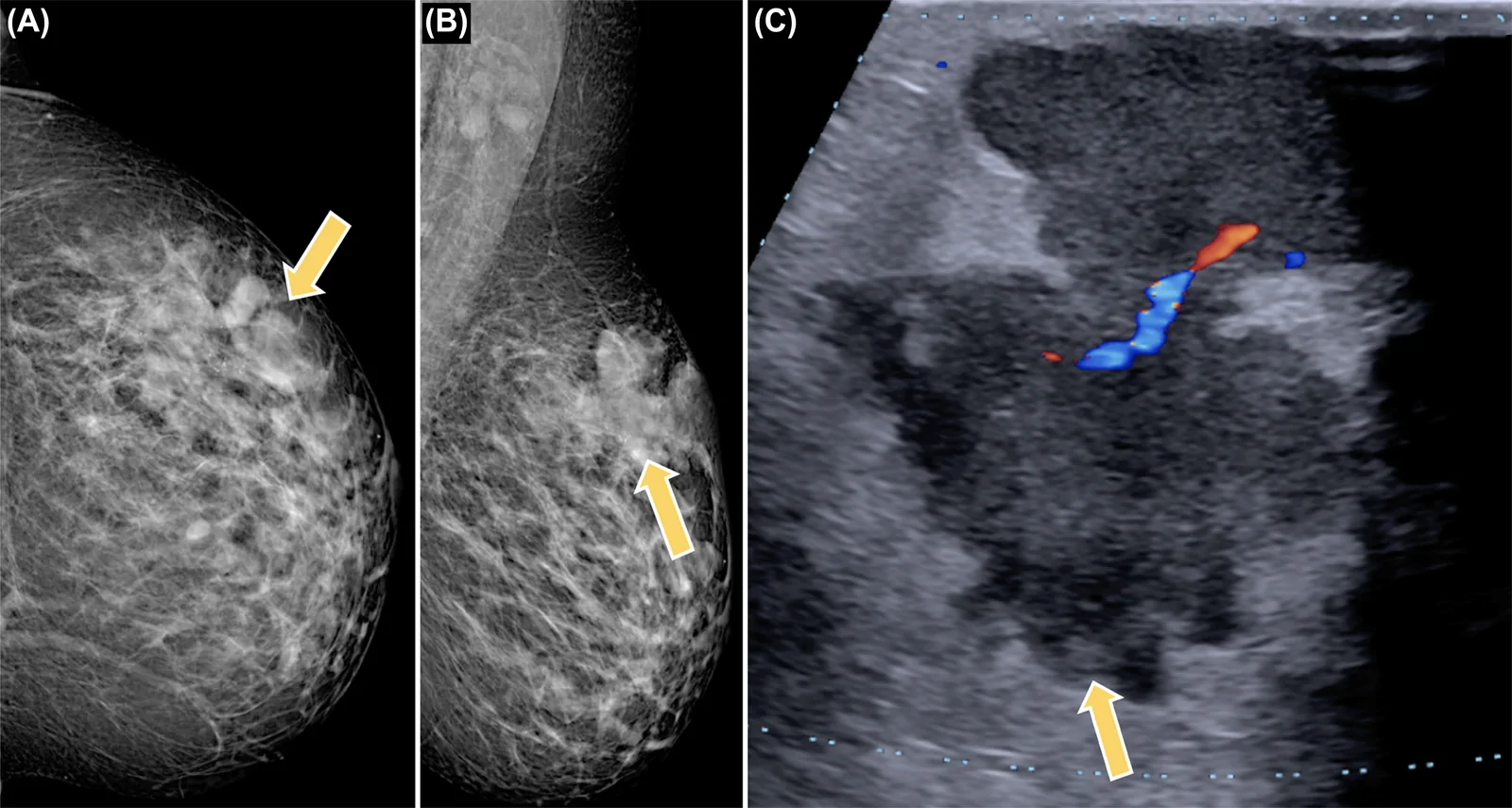
BI-RADS 4B lesion. Craniocaudal (**A**) and medio-lateral oblique (**B**) MMG views of the left breast show an irregular, microlobulated nodule located in the mid-depth of the upper outer quadrant (orange arrow), with associated architectural distortion. US (**C**) shows an irregular, non-parallel nodule with microlobulated margins and a central Doppler signal (orange arrow).

**Fig. (3):**
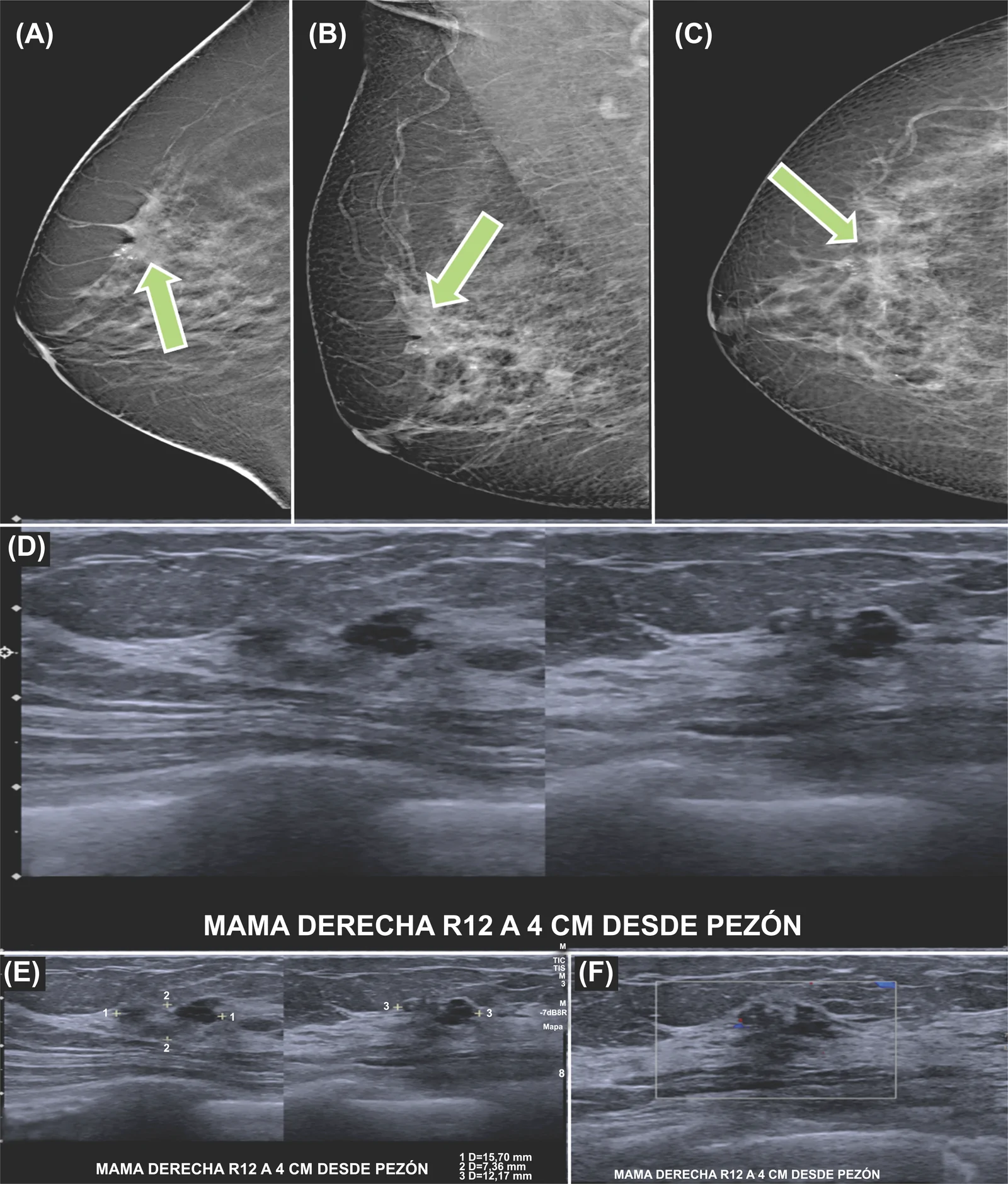
BI-RADS 4C lesion. (**A**) The right breast exhibits heterogeneously dense parenchyma with an irregular, indistinct nodule of equal density to adjacent tissue, associated with grouped amorphous calcifications and architectural distortion, located in the anterior third of the upper outer quadrant (green arrow in tomosynthesis). Medio-lateral oblique (**B**) and craniocaudal (**C**) projections confirm the findings (green arrows). US (**D**–**F**) shows an irregular, parallel, angulated-margin, heterogeneous, avascular nodule at the 12 o’clock position, 4 cm from the nipple, with posterior acoustic shadowing and enhancement, architectural distortion, and intranodular calcifications. The lesion measures 15.7 × 7.36 × 12.17 mm.

**Fig. (4):**
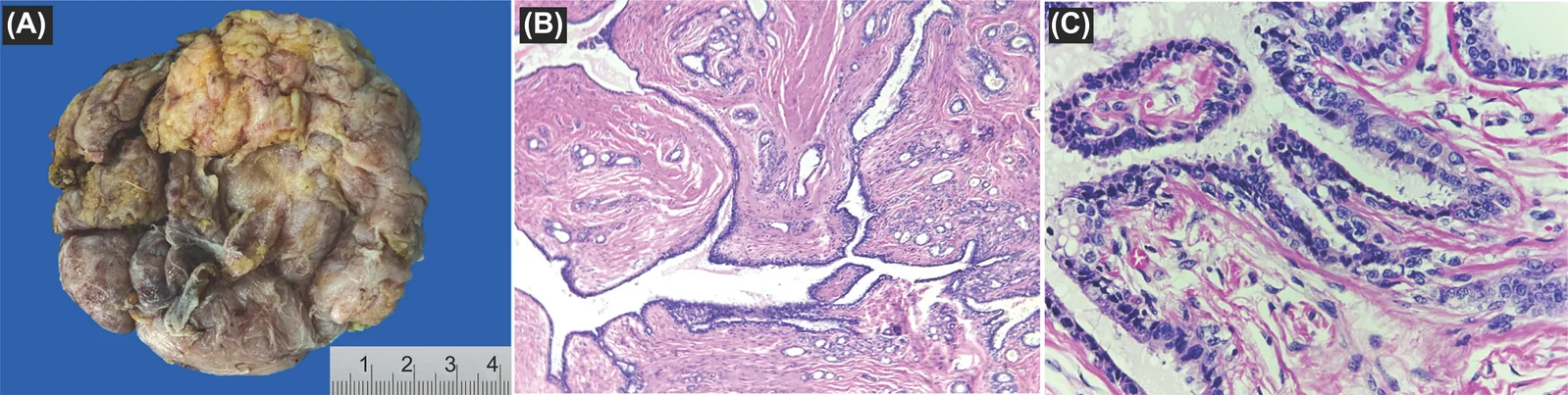
Histopathological Features of a Borderline Phyllodes Tumor (BI-RADS 4A). (**A**) Gross specimen shows a well-circumscribed, lobulated mass with a characteristic “leaf-like” growth pattern, consistent with a phyllodes tumor. (**B**) Low-power photomicrograph (hematoxylin and eosin stain) reveals biphasic architecture with cleft-like epithelial-lined spaces and stromal overgrowth. (**C**) High-power view demonstrates moderately cellular stroma with mild to moderate nuclear atypia and expansile, pushing margins, features consistent with a borderline phyllodes tumor.

**Fig. (5):**
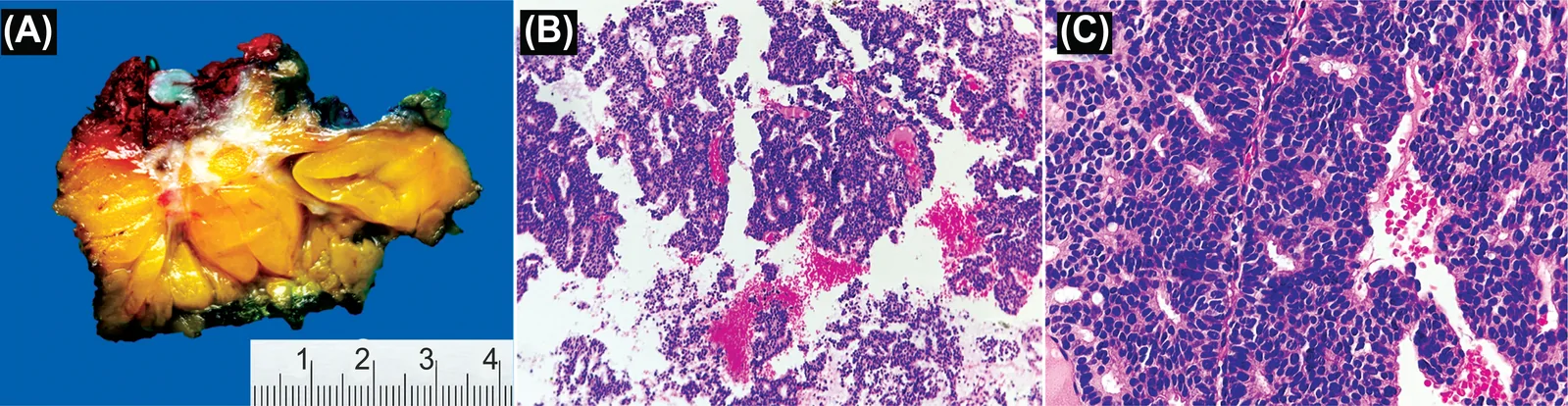
Low-Grade Invasive Ductal Carcinoma (BI-RADS 4B). (**A**) Gross section from wide local excision reveals an ill-defined, firm, gray-white lesion with infiltrative borders. (**B**) Low-power photomicrograph (H&E stain) demonstrates well-formed tubules with minimal cytological atypia, consistent with a low nuclear grade (Nottingham Grade 1). (**C**) High-power image shows regular nuclei, small nucleoli, and rare mitotic figures, hallmarks of a well-differentiated invasive ductal carcinoma.

**Fig. (6):**
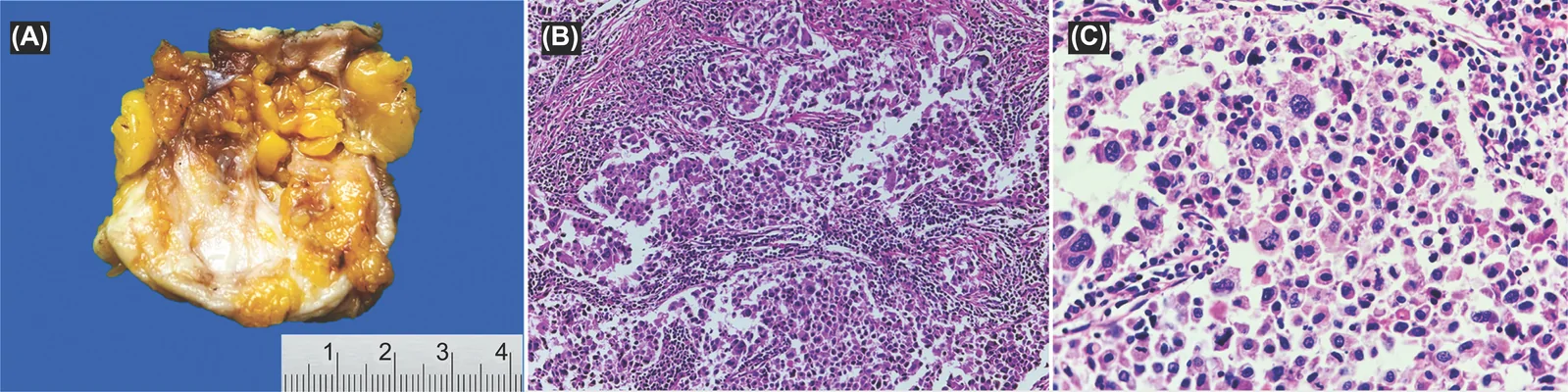
High-Grade Invasive Ductal Carcinoma (BI-RADS 4C). (**A**) Gross image reveals an irregular, poorly demarcated white lesion consistent with infiltrative carcinoma. (**B**) Low-magnification H&E section exhibits sheets of malignant cells with a solid growth pattern and limited gland formation. (**C**) High-power view shows marked nuclear pleomorphism, coarse chromatin, conspicuous nucleoli, and brisk mitotic activity, features typical of high-grade invasive ductal carcinoma.

**Table 1:** Age Distribution by BI-RADS Subcategory. This table summarizes descriptive statistics (mean, standard deviation, and age range) for patients’ ages by BI-RADS 4 subcategory. The data illustrate the increasing average Age associated with higher-risk BI-RADS classifications.

**BI-RADS Subcategory**	**Mean Age (years)**	**Standard Deviation**	**Age Range (years)**
4A	42.1	10.2	18–60
4B	47.8	12.5	25–70
4C	55.3	14.0	40–81

**Table 2:** Distribution of lesion types among BI-RADS 4 cases. This table summarizes the frequency and percentage of lesion types identified on imaging, including nodules, calcifications, lymphadenopathy, and collections.

**Lesion type**	**Frequency (n)**	**Percentage (%)**
Nodule	144	83.2
Microcalcification	5	2.9
Amorphous calcification	6	3.5
Axillary lymph node	9	5.2
Breast collection	9	5.2

**Table 3:** Histopathological Diagnoses of BI-RADS 4 Lesions. This table presents the most frequent histopathological diagnoses identified in patients with BI-RADS 4 lesions, grouped into benign and malignant categories.

**Histopathological Diagnosis**	**Frequency (n)**	**Percentage (%)**
**Benign lesions**		
Fibroadenoma (hyalinized/calcified)	33	19.1
Stromal fibrosis	28	16.2
Mastitis	14	8.1
Other benign lesions	15	8.7
**Total benign lesions**	**90**	**52.0**
**Malignant lesions**		
Ductal Carcinoma *In Situ* (DCIS)	19	11.0
**Total malignant lesions**	**19**	**11.0**

**Note:** Only the most frequent histopathological diagnoses are included. Less common or rare diagnoses were excluded from the breakdown but are reflected in the total cohort size (n = 173).

**Table 4:** BI-RADS Distribution and Malignancy Counts. This table presents the distribution of patients across BI-RADS 4 subcategories, including total case counts and the corresponding number of malignant and benign histopathological findings, illustrating the diagnostic stratification of malignancy risk.

**BI-RADS Category**	**Total Cases**	**Malignant (n, %)**	**Benign (n, %)**
4A	40	3 (7.5%)	37 (92.5%)
4B	30	12 (40.0%)	18 (60.0%)
4C	20	17 (85.0%)	3 (15.0%)

**Note:** This table includes only the patients with complete BI-RADS 4A–4C classifications and confirmed histopathological diagnoses. The remaining patients (n=83) were excluded from this analysis due to missing or non-qualifying BI-RADS subcategories.

**Table 5:** Lesion Laterality by BI-RADS Subcategory. This table displays the distribution of lesion laterality across BI-RADS 4 subcategories, reported as both frequencies and percentages. A consistent predominance of left-sided lesions is observed in all risk categories.

**BI-RADS Subcategory**	**Left Breast**	**Right Breast**	**Bilateral**
4A (n = 70)	61 (87.1%)	6 (8.6%)	3 (4.3%)
4B (n = 60)	45 (75.0%)	12 (20.0%)	3 (5.0%)
4C (n = 43)	37 (86.0%)	4 (9.3%)	2 (4.7%)

**Note:** Frequencies are based on available patient data from each BI-RADS subcategory (n = 173 total). Minor rounding adjustments may apply.

## Data Availability

The data and supportive information are available within the article.

## LIST OF ABBREVIATIONS

BI-RADS: Breast Imaging Reporting and Data System
CNB: Core Needle Biopsy
DCIS: Ductal Carcinoma In Situ
DBT: Digital Breast Tomosynthesis
FFDM: Full-Field Digital Mammography
FNA: Fine Needle Aspiration
H&E: Hematoxylin and Eosin
HGM: Hospital General de México
IDC: Invasive Ductal Carcinoma
ILC: Invasive Lobular Carcinoma
IRB: Institutional Review Board
MMG: Mammography
MRI: Magnetic Resonance Imaging
SD: Standard Deviation
SPSS: Statistical Package for the Social Sciences
STROBE: Strengthening the Reporting of Observational Studies in Epidemiology
US: Ultrasound
WHO: World Health Organization

## STANDARDS OF REPORTING

STROBE guidelines were followed.
